# Food trade among Pacific Island countries and territories: implications for food security and nutrition

**DOI:** 10.1186/s12992-022-00891-9

**Published:** 2022-12-14

**Authors:** Anne Marie Thow, Amerita Ravuvu, Siope Vakataki Ofa, Neil Andrew, Erica Reeve, Jillian Tutuo, Tom Brewer

**Affiliations:** 1 Menzies Centre for Health Policy and Economics, School of Public Health Charles Perkins Centre (D17), University of Sydney, Camperdown, NSW 2006 Australia; 2 Non Communicable Disease Program, Public Health Division, Pacific Community, Suva, Fiji; 3 United Nations Economic and Social Commission for Asia and the Pacific, Bangkok, Thailand; 4 Australian National Centre for Ocean Resources and Security, University of Wollongong, Wollongong, NSW Australia; 5 Global Centre for Preventive Health and Nutrition (GLOBE), Institute for Health Transformation, School of Health and Social Development, Deakin University, 1 Gheringhap Street, Geelong, Victoria 3220 Australia; 6 WorldFish, Honiara, Solomon Islands

**Keywords:** Trade, Nutrition, Food security, Regionalism, Food policy

## Abstract

**Background:**

There is growing attention to intra-regional trade in food. However, the relationship between such trade and food and nutrition is understudied. In this paper, we present an analysis of intra-regional food trade in the Pacific region, where there are major concerns regarding the nutritional implications of international food trade. Using a new regional database, we examine trends in food trade among Pacific Island Counties and Territories (PICTs) relative to extra-regional trade.

**Results:**

Intra-regional trade represents a small, but increasing proportion of total imports. The major food group traded within the Pacific is cereal grains and flour, which represented 51% of total intra-regional food trade in 2018. Processed and prepared foods, sweetened or flavoured beverages, processed fish, and sugar and confectionary are also traded in large quantities among PICTs. Trade in root crops is negligible, and overall intra-regional trade of healthy foods is limited, both in terms of tonnage and relative to imports from outside the region. Fiji remains the main source of intra-regional imports into PICTs, particularly for non-traditional staple foods.

**Conclusions:**

This study highlights the growth in trade of staple foods intra-regionally, indicating a role for Fiji (in particular) in regional food security. Within this overall pattern, there is considerable opportunity to enhance intra-regional trade in traditional staple foods, namely root crops. Looking forward, the current food system disruption arising from the COVID-19 pandemic and associated policy measures has highlighted the long-term lack of investment in agriculture, and suggests an increased role for regional approaches in fostering trade in healthy foods.

**Supplementary Information:**

The online version contains supplementary material available at 10.1186/s12992-022-00891-9.

## Introduction

Global attention has turned to regional trade as multilateral negotiations have continued to stall over the past 20 years. Intra-regional trade agreements can create new opportunities for specialization and comparative advantage, and open proximal markets through reduced barriers to trade (both tariff and non-tariff) and enhanced regional stability [1]. For developing countries, intra-regional trade agreements can also foster economic stability and enhanced capacity to engage with external trade partners [2, 3]. Broadly, drivers of regionalism include expected gains arising from reduced transaction costs, shared innovation, and greater economic and political weight in international markets and institutions [4].

The relationship between intra-regional trade and food and nutrition security is complex and dependent on regional and country-level characteristics. As a consequence, the degree to which intra-regional food trade contributes to the United Nations Sustainable Development Goals (notably 1 to 3) is contingent on the details of that trade [5]; despite potential advantages, barriers to intra-regional trade in food remain [6]. There have been recommendations for deeper regional integration to enable freer flows of food from surplus to deficit countries to address food insecurity [7]. Indeed, intra-regional trade cooperation in the Association of South East Asian Nations has improved food security through staple food trade [8], as has the South Asian Free Trade Agreement, in the form of regionalised meat trade [9]. Conversely, regional liberalization has also been associated with the nutrition transition in Central America [10, 11] and Southern Africa [12].

The food security impacts of COVID-19 have also highlighted the vulnerability of net food importing countries to food insecurity. Studies of regional trade within Africa have highlighted the potential for regional trade to mitigate this vulnerability [8], as well as the potential for COVID-19 associated disruptions to stimulate regional food markets and reduce reliance on global trade [13]. Similar opportunities have been observed in the Pacific region [14]. In particular, local agricultural production and incomes have reduced, as domestic markets have declined and access to international markets has been disrupted. However, this has also led to a resurgence in traditional food systems [15].

Concerns about trade and the nutrition transition – in which diets globally have shifted towards increased intakes of more processed foods high in fat, salt and sugar, with low intakes of fruit, vegetables and fibre [16] – have been prominent for Pacific Island Countries and Territories (PICTs) [17].^1^ The region is one of the most affected by diet-related non-communicable diseases globally, and also faces persistent food insecurity (often as a result of natural disasters) [18, 19]. Historically, food trade in the Pacific has been strongly influenced by colonization and extra-regional trade [20]. Rising import dependence has generated concerns about the dumping of unhealthy products in the region and the impact of colonial trade patterns, and the role of imports in fostering dietary change [20, 21]. There has been a marked change in diets from consisting of mainly healthy traditional, local foods – including root crops, fish, and vegetables – to diets including a range of non-traditional, often imported foods, such as rice, sugar, wheat flour and processed snack foods [22].

While Pacific Island leaders recognize the importance of trade policy in improving nutrition in the region [23], there appears to be an implicit assumption that intra-regional trade is small and of little importance [24]. There have also been concerns raised regarding the limited potential benefits (and potential costs) of regional economic integration and intra-regional trade in the face of intractable barriers such as the high costs of transportation, limited production capacity and small market size [25–27].

In this study, we provide a critical and missing piece of evidence in analysing regional food trade policy in the Pacific Island region by quantifying trends in intra-regional trade, addressing the following research questions: 1) is intra-regional trade significant in terms of food security and nutrition?, and 2) have regional trade agreements contributed to the level of intra-regional trade?. We present findings from an analysis of the recently developed Pacific Food Trade Database (PFTD), informed by nutritional considerations consistent with global approaches to trade and nutrition analysis [28, 29]. We highlight the central role Fiji plays as a regional export and re-export hub in the region [30, 31]. Further, we provide a first critical analysis of the impact of the Pacific Island Countries Trade Agreement (PICTA) as a major regional food trade policy instrument.

### Background on intra-regional trade in Pacific Island countries and territories

The first intra-regional agreement signed among Pacific countries was the Melanesian Spearhead Group (MSG) trade agreement in 1993, between Papua New Guinea, Solomon Islands and Vanuatu, with Fiji joining in 1996 [32]. In relation to food trade, the parties committed to eliminating duties and other restrictions, including providing exemptions on import duties for meat, fish, oils, noodles, baked goods originating in these countries. In 2001, PICTA was signed by the Cook Islands, Federated States of Micronesia, Fiji, Kiribati, Niue, Samoa, Solomon Islands, Tuvalu, Vanuatu, Nauru, Papua New Guinea and Tonga. PICTA was implemented in 2007 by Cook Islands, Federated States of Micronesia, Fiji, Kiribati, Niue, Samoa, Solomon Islands, Tuvalu and Vanuatu, and included reductions in tariff rates for most food items, albeit with fairly significant exemptions on the part of Kiribati, Niue, Papua New Guinea, Solomon Islands, Tuvalu and Vanuatu. PICTA was also envisaged by the Parties as the first step to deeper regional integration, including a common market. A treaty establishing the Micronesian Trade and Economic Community (MTEC) was concluded in 2014, including Federated States of Micronesia, Marshall Islands, and Palau, but there are no specific commitments to intra-regional trade liberalization.

Pacific Island governments envisage both political and economic benefits from regional integration, including strengthening domestic commitment across the region to liberalization, attracting development, providing a single voice in international fora, enlarging the market size, and providing a gradual adjustment towards more significant (extra-regional) liberalization [26]. Intraregional trade agreements have also played an important role in trade facilitation in the Pacific [33] and facilitated sectoral cooperation and regional service delivery [34]. The overall impact of intra-regional agreements on trade, however, has been limited. There has been some growth in intra-regional trade flows, but due to high costs, limited markets, a lack of strategic investment in agriculture and manufacturing, and bureaucratic regulations, extra-regional trade remains dominant [27, 35].

## Methods

### Study design

We conducted a descriptive quantitative analyses of existing regional trade data. A number of the analyses are comparative in nature in that we compare between, for example, temporal trends in quantity of food traded between countries and food types. Data include food and beverage commodities, countries, quantities and year in which trade occurred. Interpretation of the results was conducted by the authors, whose Pacific-focused trade expertise spans nutrition, policy, and trade value chains.

### Data source

To characterize intra-regional trade of food and beverages (hereafter, unless specified otherwise ‘food’ is used as shorthand for ‘food and beverages’) in the Pacific we use the Pacific Food Trade Database (PFTD) [36]. The PFTD is derived from the BACI HS92 global trade database of international commodity trade [37] which uses United Nations Comtrade data as its primary and only data source. The PFTD is the result of extensive cleaning of the BACI data on food trade relevant to Pacific Island Countries and Territories (PICTs). The PFTD includes all food trade flows at subheading level across 18 PICTs for the years 1995–2018.

Some commodities were excluded because they fell outside the scope of the analysis: still and carbonated water, tobacco and alcohol. Tuna (except canned) was excluded due to ongoing concerns relating to data quality for these commodities (other types of fish and invertebrates are included). Coconuts (HS080110) were removed from analyses relating specifically to nuts, as a healthy source of food, due to the large and variable trade volumes which suggests copra has periodically been mis-classified as coconuts. It was retained elsewhere, as was copra (HS120300) because some derivatives of copra are used for human consumption and it represents a major export cash crop for many PICTs including significant intra-regional trade.

### Analysis

First, we present an overview of intra-regional trade relative to imports and exports with the rest of the world, and provide coarse analysis of the Pacific countries that dominate intra-regional trade. Second, we explore the temporal trends in quantity of the different types of food traded within the region. Foods were grouped with reference to commodity types and the Pacific Food Guide [38]. Third, we explore intra-regional trade of staples, and healthy and unhealthy food as key dimensions of food security and diets. The assessment of healthy and unhealthy foods used the INFORMAS classification [29]. This framework was chosen for its relevance to the context of this study following Ravuvu, Friel [39].

Fourth, we determine whether PICTA had any measurable impact on either intra-regional trade or on imports from outside the region. To make this determination we compare temporal trends in quantities of food being imported from outside the region with quantities being traded within the region, across both all PICTs and PICTs that were early adopters of PICTA. Early PICTA adopters were Cook Islands, Fiji, Niue, Samoa, Solomon Islands, Tuvalu and Vanuatu. Commodities included in analysis of imports from outside the region included only those commodities that are not produced within the region (Supplementary materials 1 [see end of text]). Commodities included in analysis of trade between PICTs include only those commodities that are produced within the region. A total of 145 commodities were included as being imported from outside the region, while a total of only 9 commodities were included as only being produced and traded within the region. This commodity distinction was necessary to control for effects of retrading (foods imported from outside the region and then exported to other PICTs with no or minimal further processing). In some instances the quantities were normalised across commodities to control for within-commodity quantity variability. Data were normalised over the range of 0 to 1 as:

1 $$normalised\ value=\frac{X_i-{X}_{min}}{X_{max}-{X}_{min}}$$

Where *X*_*min*_ and *X*_*max*_ are the smallest and largest trade quantities of the commodity reported from 1995 to 2018, and *X*_*i*_. is the trade quantity to be normalised.

## Results

### Overview of intra-regional food trade

**Fig. 1:**
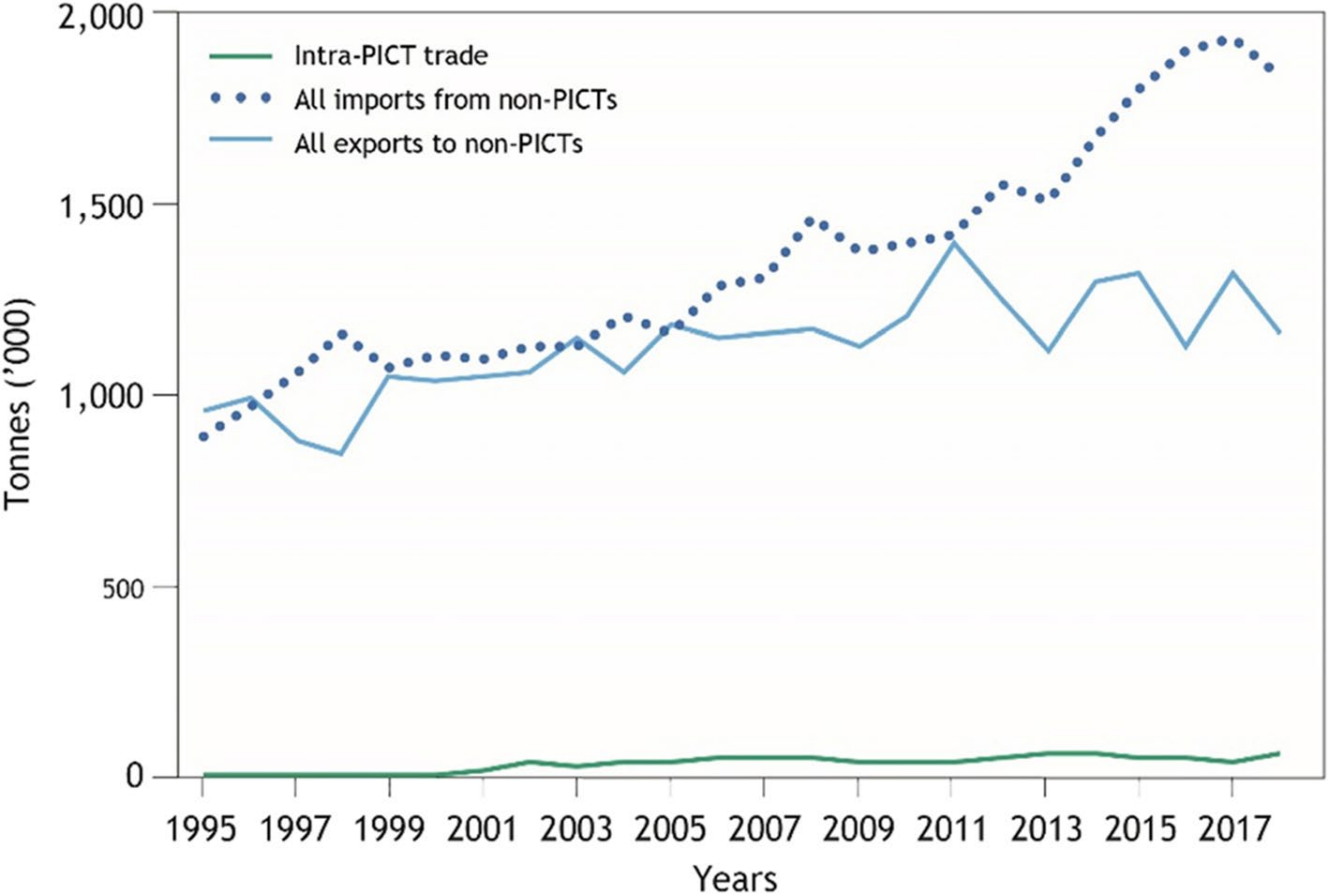
Intra-regional food trade compared to extra-regional trade (excluding alcoholic beverages, tuna, and water), 1995–2018

**Fig. 2:**
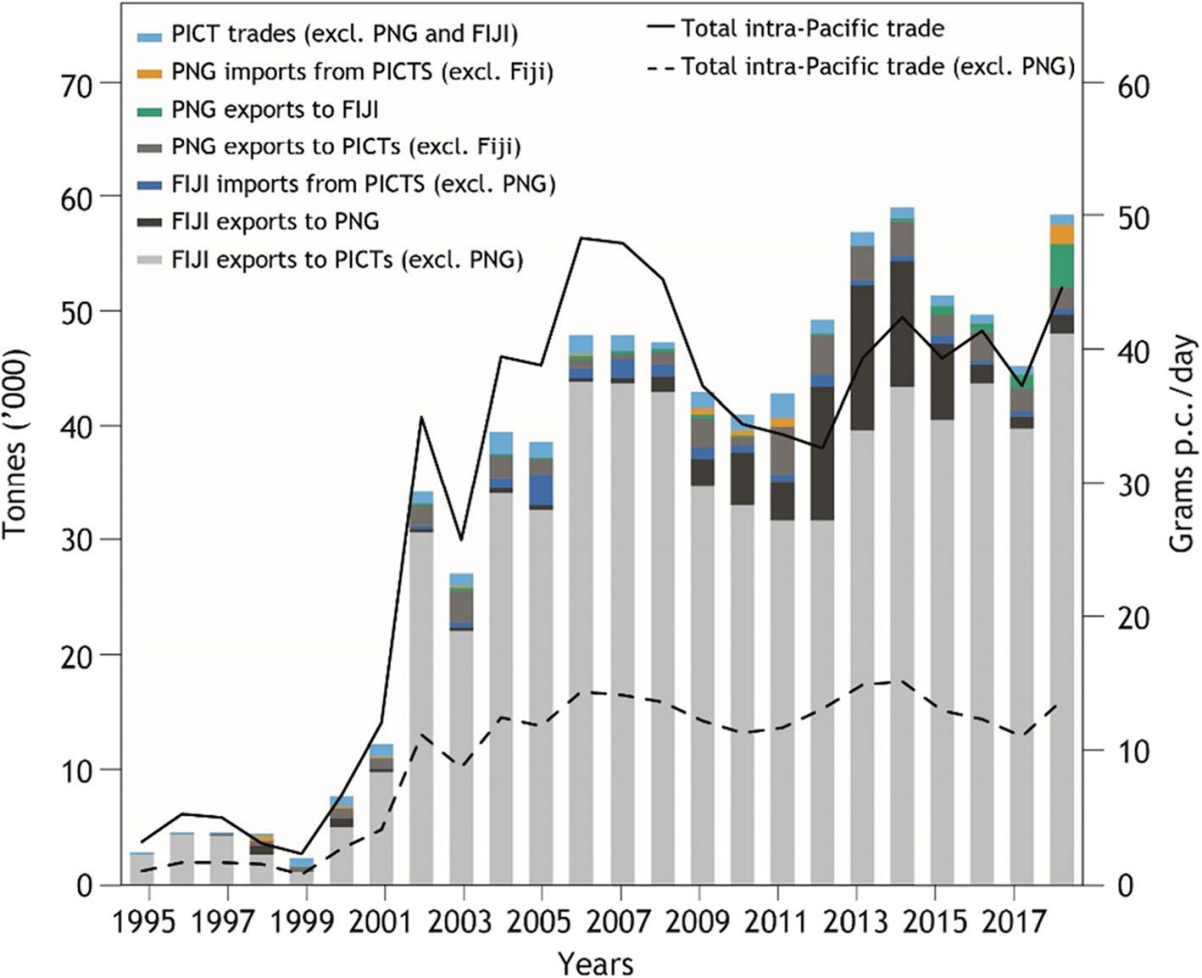
Intra-regional food trade in the Pacific Region, 1995–2018. Bars show cumulative total trade across different PICT combinations. Lines indicate per capita trade flows with PNG (solid) and without (dashed)

Intra-regional food trade represents only a small fraction of total food imported by PICTs: rising from 0.3% in 1995 to 3.2% in 2018 (Fig. 1). In 2018, 58,712 t of food was traded intra-regionally. This was a substantial increase from the 2724 t traded intra-regionally in 1995, with the major increase occurring between 2000 (7819 t traded) and 2001 (12,325 t traded) (Fig. 2). Food trade between PICTs and non-PICTs has been dominated by Australia and New Zealand. The bulk of exports from the region are comprised of sugar and palm oil.

Fiji has consistently been the main source of intra-regional imports to PICTs, with the volume rising from 2693 t (99% of total) in 1995 to 49,900 t (85%) in 2018 (Fig. 2). Countries importing the most food from Fiji, on a per capita basis, include Cook Islands, Kiribati, Nauru, Samoa, Tonga, Tuvalu and Wallis and Futuna Islands. Exports from Papua New Guinea to other PICTs have increased from a negligible amount to nearly 10% of intra-regional food trade.

Papua New Guinea has the largest population in the region, and was the single largest destination for intra-regionally traded food from 2012 to 2014 (18–24% of trade). However, Papua New Guinea represents by far the lowest per capita consumption of intra-regionally traded foods, even during this period. In 2018, total per capita consumption of intra-regionally traded foods was 14 g/capita/ day, but excluding Papua New Guinea this rises to 45 g/capita/day (Fig. 2).

### Types of food traded intra-regionally

**Fig. 3:**
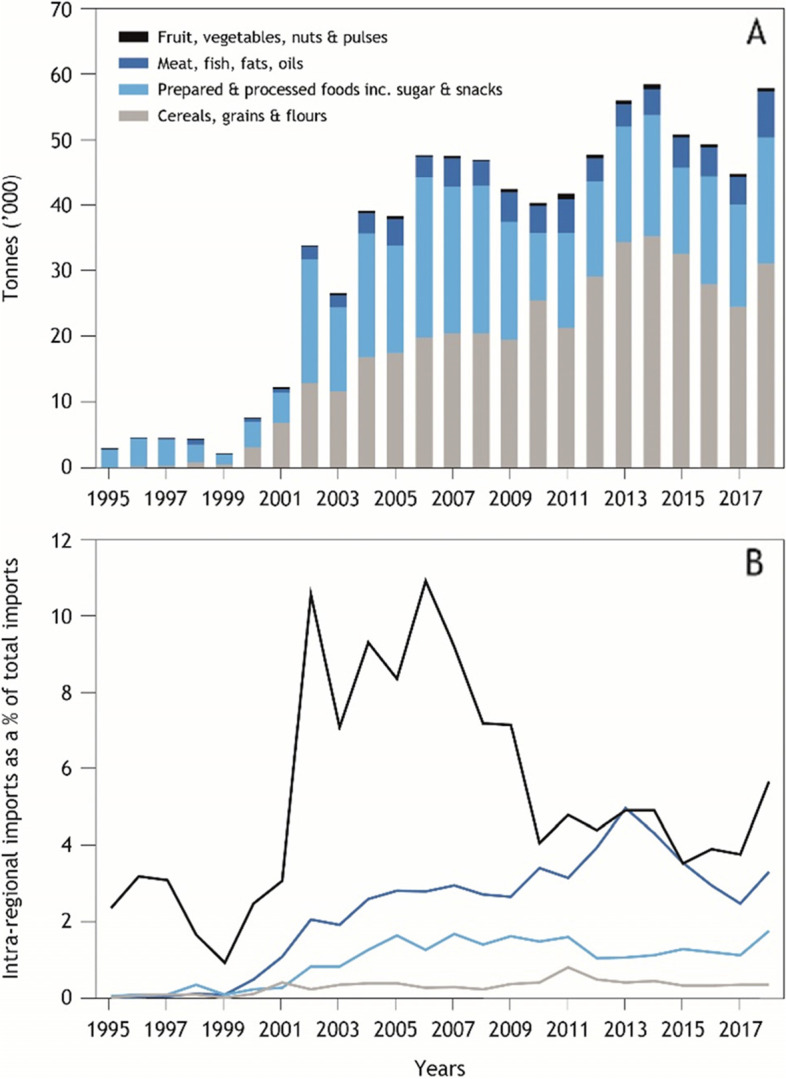
Intra-regional trade by food groups including **A** tonnes imported to PICTs from other PICTs and **B** percentage of all imports that are imported from PICTs

The major food group traded intra-regionally in the Pacific is cereals, grains and flours, which represented 51% of total intra-regional food trade in 2018 (Fig. 3), 98% of which is exported from Fiji to smaller Pacific island countries. The major cereal traded in 2018 was wheat flour, at 84% of cereal, grains and flour trade and 45% of total intra-regional food trade. Rice comprised only 1% of cereal, grains and flours traded and 0.6% of total intra-regional food trade (nearly all rice is directly imported from outside the region). Cereal grains and rice are all imported from outside the region, except for limited rice production in Fiji, and are either re-traded or milled and exported as flour and milled rice.

Trade in processed and prepared foods, including processed meat, vegetable and ‘miscellaneous’ preparations, comprised 19% of intra-regional food trade in 2018. The next highest trade food groups were sweetened or flavoured beverages (8%), processed fish (5%) and sugar and confectionary (3%). Trade in root crops is negligible, totalling 78 t in 2018.

Intra-regional trade represents a small, but increasing proportion of total imports (Fig. 3). The most notable increases have been in intra-regional imports of ‘cereals, grains and flours’, which also rose as a proportion of total imports (from negligible in 1999 to over 4% in 2013 before declining to 2–3%). The large spike in prepared and processed foods as a proportion of total imports between 2001 and 2007 reflects the sustained increase in sugar export from Fiji to other PICTs that occurred in 2001.

### Intra-regional trade in staple foods

Intra-regional trade of the major ‘non-traditional’ staple foods (namely rice and wheat flour) represented 3% of total trade in these foods during the period 2014–2018 (Table 1). Fifty-one percent and 26% of total trade in staple foods went to Papua New Guinea and Fiji respectively during this period. Almost all of this was imported from countries outside of the Pacific region. During this period, 2062 t of staple foods were imported into Fiji from Papua New Guinea between 2014 and 2018 (the only intra-regional export from Papua New Guinea), representing only 0.2% of staple food imports into Fiji.

**Table 1:**
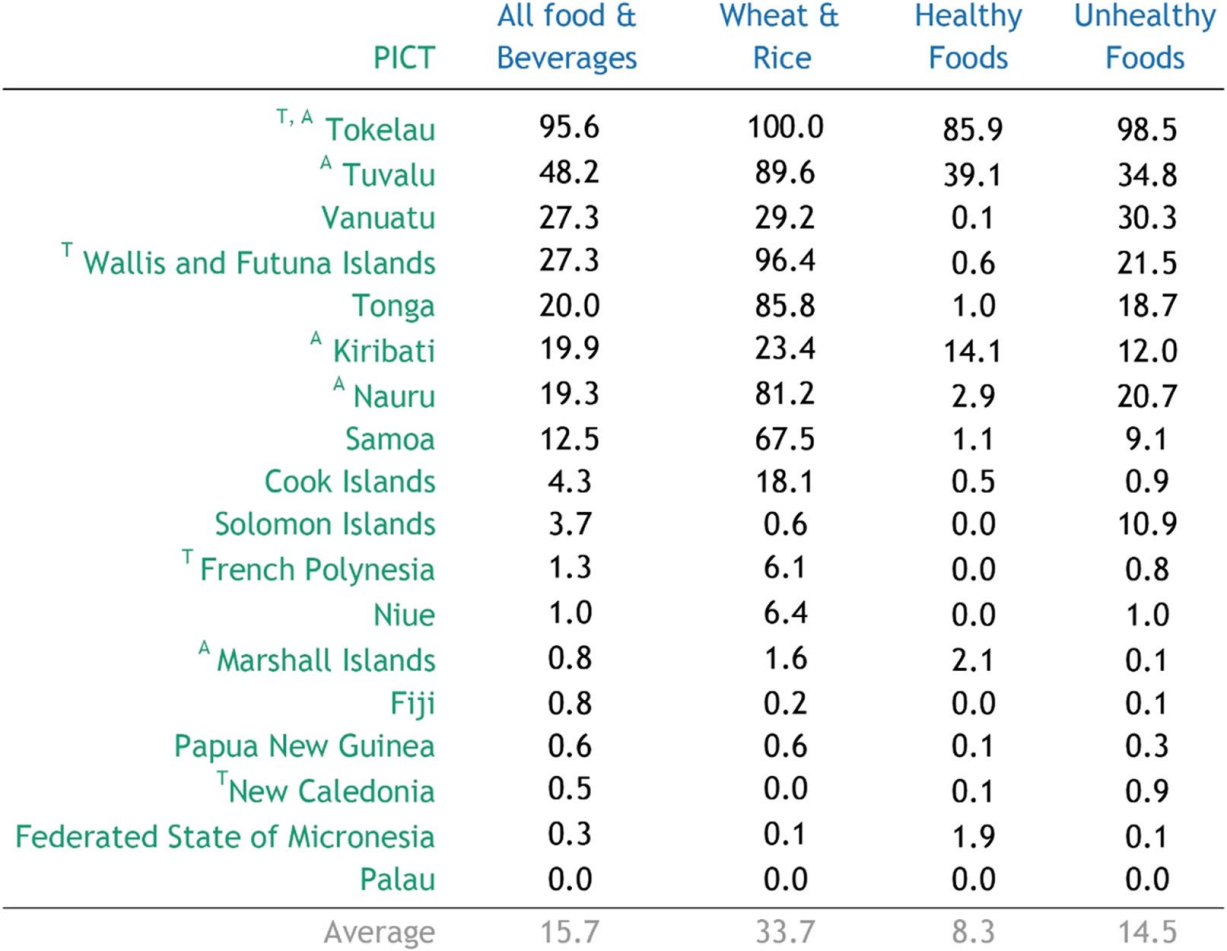
Intra-regional imports as a percentage of total imports (by weight; 2014–2018 avg)

^***T***^ Overseas Territory, ^*A*^ Atoll nation, defined as the capital city being on an atoll island, rather than a high island. Category definitions (see methods for details): Wheat and Rice includes all of HS1001, HS1006, and HS1101; Healthy food category includes: fruit, vegetables, pulses, nuts, seeds and root crops; Unhealthy food category includes: Sugars and other caloric sweeteners, fatty meats, savoury ready-to-eat snack foods and meals, sweet snack foods and energy dense beverages

The significance of intra-regional trade in rice and wheat varied widely across countries during the period 2014–2018. For Nauru, Tokelau, Tonga, Tuvalu and Wallis and Futuna Islands, intra-regional trade comprised more than 80% of total non-traditional staple food imports (Table 1). In contrast, intra-regional trade contributed less than 1% of imported non-traditional staple foods for the Federated States of Micronesia, Fiji, New Caledonia, Papua New Guinea and Solomon Islands.

**Fig. 4:**
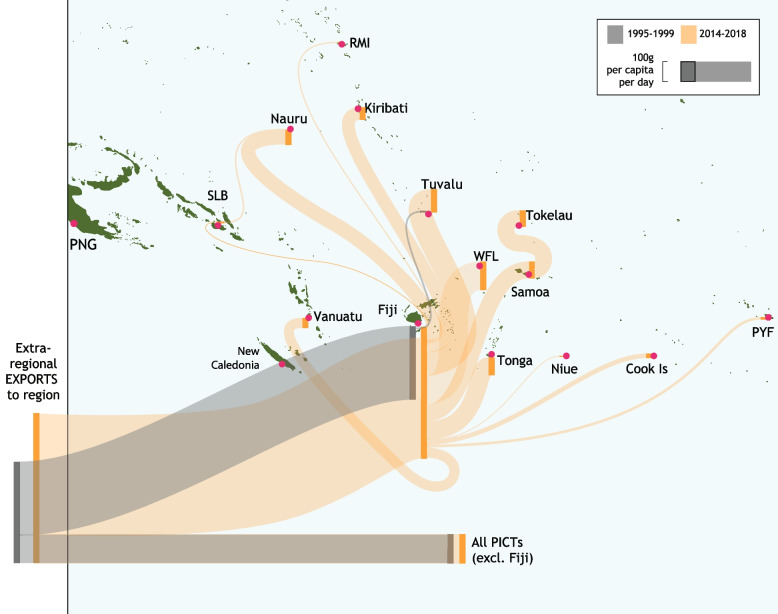
Average annual grams per capita per day of staples (HS10, HS11) moving between PICTs and imports from outside the region for 1995–1999 and 2014–2018. Line width reflects grams per capita per day for the importing country (see scale bar). Per capita imports entering the region to countries other than Fiji are aggregated because there is negligible re-trade from PICTs other than Fiji

Fiji acts as a hub for intra-regional trade in staple foods, with 98% of intra-regional trade in non-traditional staple foods coming from Fiji (Fig. 4). Fiji was the source of all intra-regional non-traditional staple food imports into Cook Islands, Federated State of Micronesia, French Polynesia, Kiribati, Marshall Islands, Nauru, New Caledonia, Niue, Papua New Guinea, Samoa, Solomon Islands, Tonga, Tuvalu, Vanuatu and Wallis and Futuna Islands. For Nauru, Samoa, Tonga, Tuvalu and Wallis and Futuna Islands, over 60% of total non-traditional staple food imports came from Fiji. An average of 895,143 t of staples entered the region per year between 2014 and 2018, 237,243 t of which was imported by Fiji; 177 g/capita/day was imported directly from outside the region to other PICTs. Of Fiji’s imports roughly 11% was reexported, or processed and exported, to other PICTs.

### Intra-regional trade of healthy foods

**Fig. 5:**
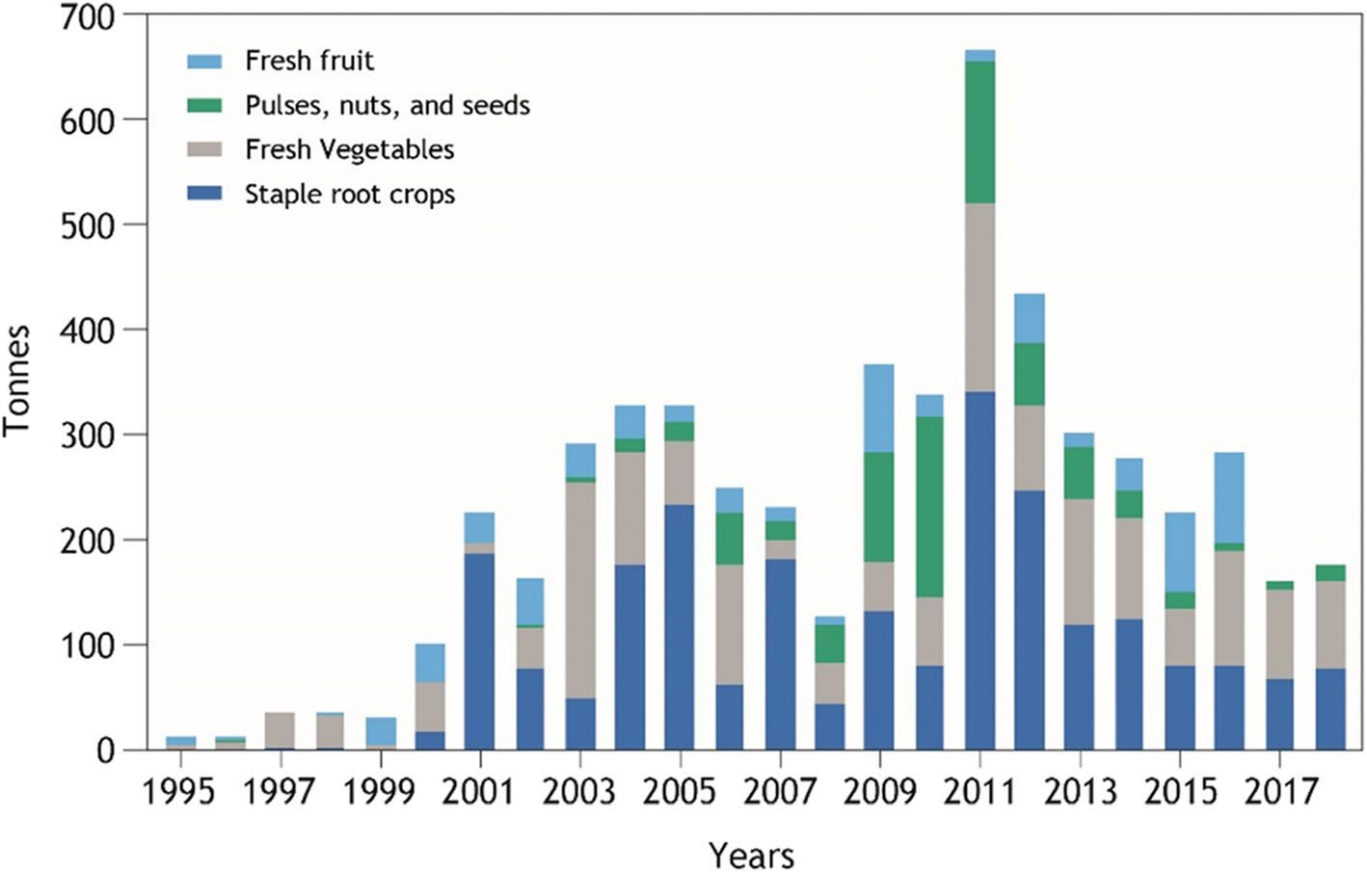
Total intra-regional trade in healthy food, subdivided across four healthy food groups

Intra-regional trade of healthy foods, including fruit, vegetables, pulses, nuts and seeds, and root crops, is limited, both in terms of tonnage and relative to imports from outside the region (Fig. 5). The peak volume of intra-regional trade in healthy foods was 656 t in 2011.In 2018, healthy foods represented 0.3% of intra-regional trade, and 0.18% of total healthy food imports. The main extra-regional source country for healthy foods is New Zealand which exports a meaningful quantity of vegetables to the region.

Fiji was the source of 63% of healthy foods traded intra-regionally in 2018, a lower percentage than its contribution to total intra-regional food trade (85%) in the same year. The larger trade flows of staple root crops in 2011 and 2012 are cassava (HS071490 Manioc, arrowroot, sago pith etc.) from Solomon Islands to Kiribati. All intra-regional trade flows of staple root crops (2350 t) through the period are exported from high islands, and 75% of this volume is imported by atoll nations. The vast majority of healthy food imports come from outside the region. Only around 0.2% of healthy food imports were from PICTs in 2018. Fiji imports 139 g/capita/day from outside the region, mostly comprising potatoes from New Zealand. The rest of the region, excluding Fiji, directly imports only 11 g/capita/day from outside the region. Small quantities are exported from Fiji to other PICTs, predominantly atoll nations.

### Intra-regional trade of unhealthy foods

**Fig. 6:**
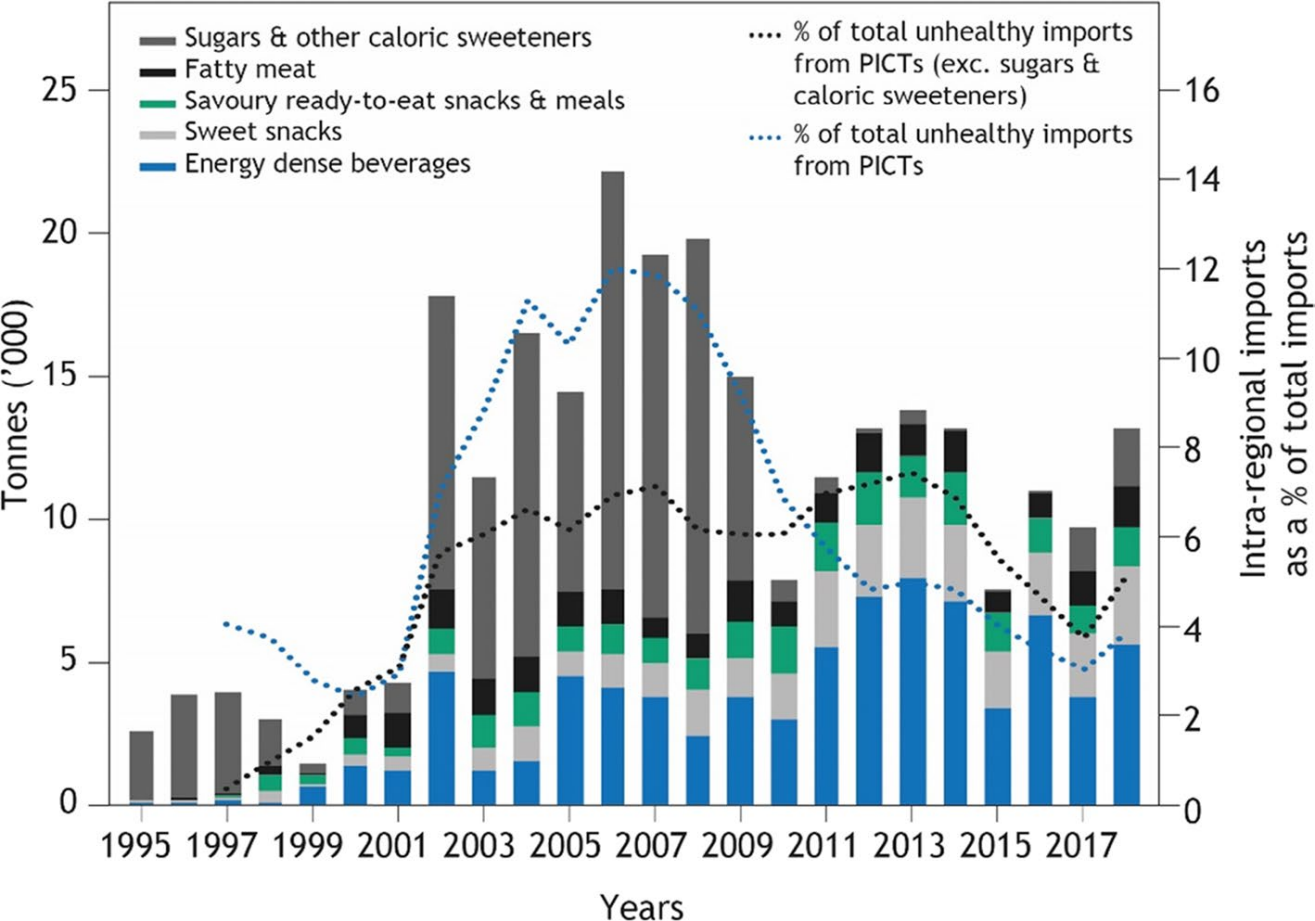
Total intra-regional trade in unhealthy food, subdivided across five food groups. Lines (z axis) shows 3 year moving average of intra-regional trade in unhealthy food as a percentage of total unhealthy food imports, with the remainder coming from outside the region

Intra-regional trade in unhealthy foods – namely sugars, fatty meats, ready-to-eat snacks and meals, sweet snacks and energy dense beverages – peaked between 2002 and 2009, at an average of nearly 16,000 t traded per year (Fig. 6). Between 2014 and 2018 the yearly average was less than 10,000 t. Intra-regional trade in sweet snacks increased from 30 t in 1995 to over 2000 t in 2011 and has remained fairly steady since. Intra-regional trade in sweetened beverages increased from 17 t in 1995 to nearly 4000 t in 2002, and then remained between 4000 and 6000 t through to 2018. The overall decline is mainly due to a decline in sugar trade, which fell from an average of over 10,000 t per year to less than 1000 t per year over the same period. In particular, Fiji as the main intra-regional exporter of sugar to PICTs, ended its preferential export price on sugar (which was two to three times higher than the world market price) in December 2007 due to the European Union reform on its Common Agricultural Policy [40]. As a result, the local sugar industry in Fiji started to face stiff competition from more efficient sugar exporters worldwide from 2008 onwards.

Overall, intra-regional trade comprised 4% of total unhealthy food trade, and for eight of the 17 countries that imported unhealthy food from within the region (the only country in our study that only had extra-regional sources of unhealthy food imports was Palau), intraregional trade comprised 1% or less of total unhealthy food imports (Fig. 5). However, for Tokelau, intra-regional trade was the source of 98% of unhealthy food imports. For Nauru, Tuvalu, Vanuatu and Wallis and Futuna, intra-regional trade comprised over 20% of total unhealthy food imports.

**Fig. 7:**
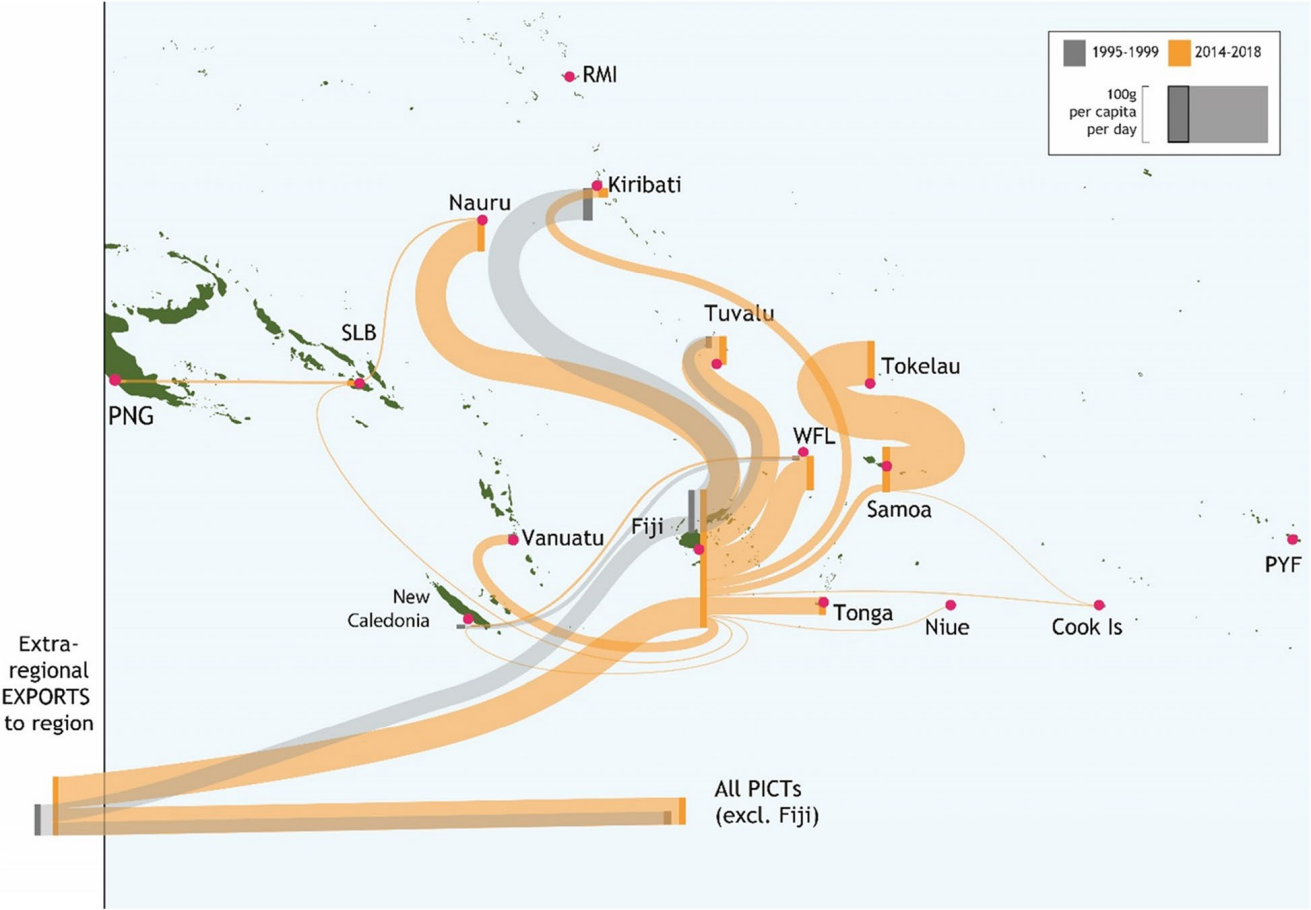
Average annual grams per capita per day of unhealthy food moving between PICTs and imports from outside the region for 1995–1999 and 2014–2018. Line width reflects grams per capita per day for the importing country (see scale bar). Per capita imports entering the region to countries other than Fiji are aggregated because there is negligible re-trade from PICTs other than Fiji

In the late 1990s, Fiji was a hub for intra-regional trade in unhealthy foods, although the volume was minimal (Fig. 7). Between 2014 and 2018 the majority of intra-regional trade in unhealthy foods came from Fiji, but with more diversity in source than observed for staple foods. Only for Federated States of Micronesia, Marshall Islands, Niue, Samoa, Tonga, Tuvalu and Vanuatu was Fiji the source of 100% of intra-regional imports. Other source countries included Papua New Guinea, Samoa, New Caledonia, Solomon Islands, Vanuatu, French Polynesia and Marshall Islands. On a grams per capita basis Fiji is a major reexport and export hub for unhealthy food in the region.

### Intra-regional trade and trade agreements

**Fig. 8:**
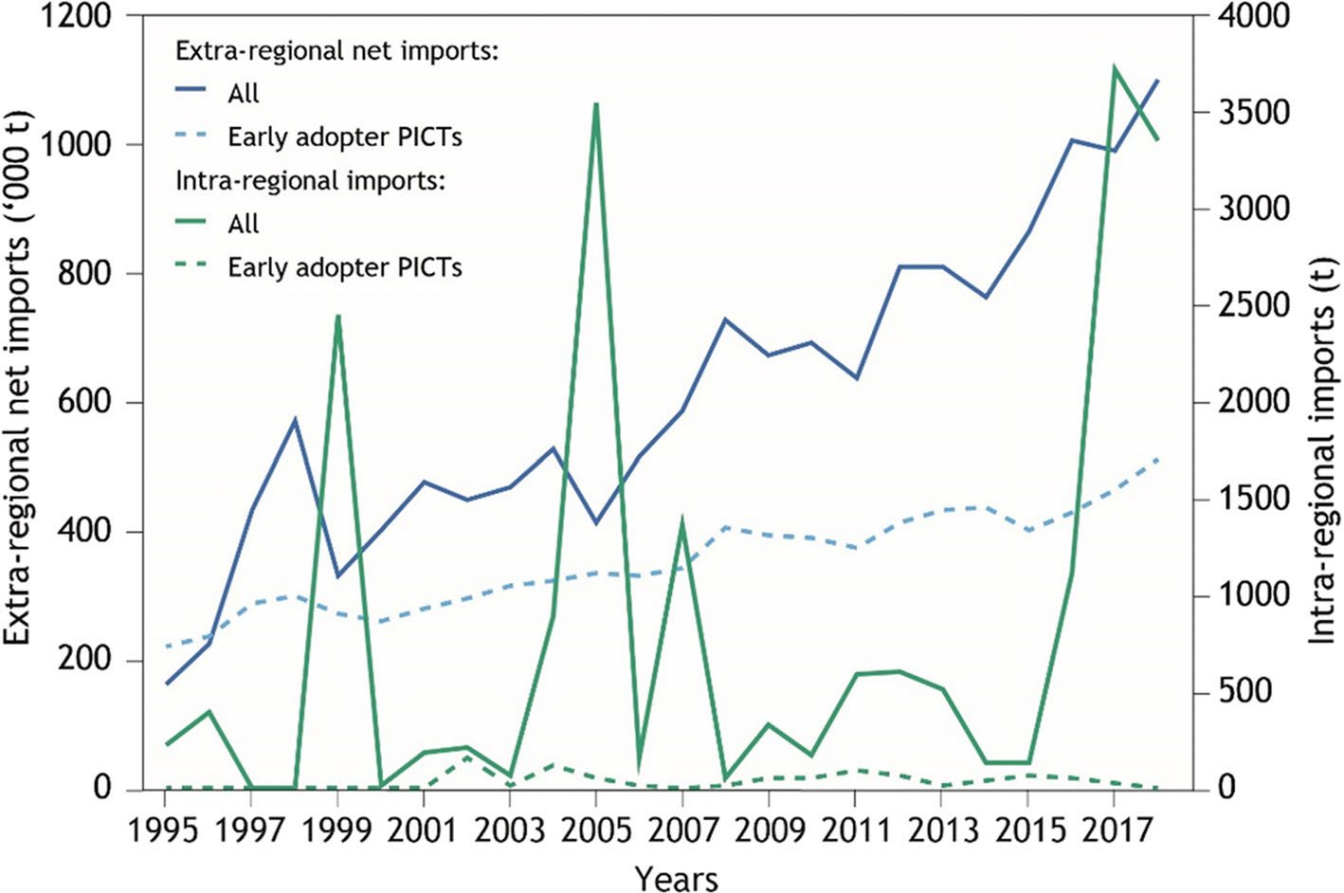
Temporal trend in quantity of food traded including net imports from outside the region to all PICTs (solid blue line) from outside the region to PICTs that were early adopters of PICTA (dashed blue line), all intra-regional imports (solid green line) and intra-regional imports for early adopters of PICTA (dashed green line)

The PICTA was signed (2001) and implemented (2007) during the period under analysis. Intra-regional imports grew substantially during this period, as described above. Here, we explore whether there was any corresponding shift in the proportion of imports sourced intra-regionally compared to extra-regionally. PICTs have adopted PICTA at different times, and to differing degrees [41]. To control for ambiguity we include the following PICTs in the PICTA only analysis as they were unambiguously early adopters to PICTA; Cook Islands, Fiji, Niue, Samoa, Solomon Islands, Tuvalu and Vanuatu. Extra-regional imports to all PICTs and to PICTA early adopters have increased considerably and consistently since the mid 1990s (Fig. 8). Trade of regionally produced commodities (Supplementary Table 1) between all PICTs has been highly variable, with temporal spikes attributed to large intra-regional shipments of copra. Trade of regionally produced commodities between early PICTA adopters has been negligible through the time period. In particular, there is no aggregate evidence of an effect of PICTA on the quantity of food trade.

**Fig. 9:**
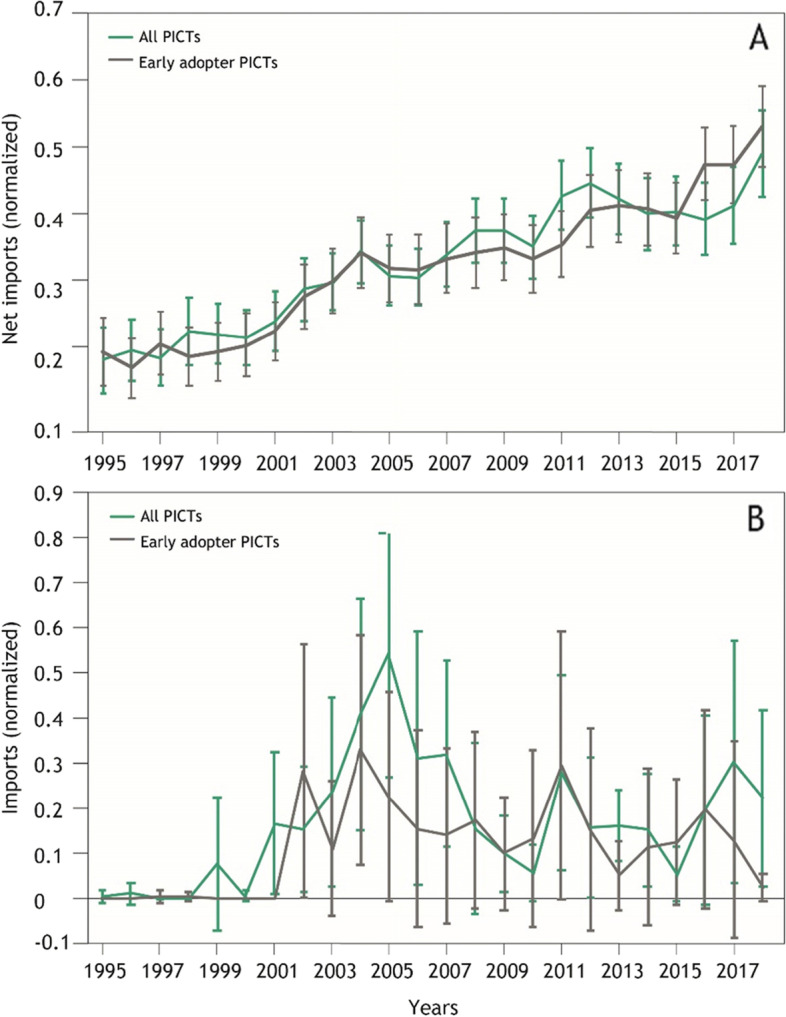
**A** Mean food imports from outside the region to all PICTs and to PICTs that were early adopters of PICTA. Only commodities produced outside the region were included; **B** Mean intra-regional trade between all PICTs and between PICTs that were early adopters of PICTA. Only commodities that were produced within the region were included. For both graphs, each HS6 commodity type was given equal weighting to avoid bulk commodities dominating trends (see methods for calculation). Error bars show 95% confidence interval around the average trade quantity. See Supplementary Table 1 for inclusion and exclusions

When controlling for the difference in quantity of different commodities there is no significant change in quantity being imported from outside the region for either all PICTs or early PICTA adopters (Fig. 9A). If PICTA had a meaningful effect on imports from outside the region we would have expected to observe some difference between the two trends. Similarly, there is no discernible difference in the quantity of Pacific produce traded between all PICTs and between early PICTA adopters (Fig. 9B). There is some increase for both trends in the early 2000s, but the increase is ephemeral and not unambiguously attributable to PICTA.

## Discussion

Intra-regional food trade among PICTs has grown and is a significant source of traded food for many countries. Fiji acts as a regional hub, with the majority of intra-regional food imports originating in Fiji. We found a heavy reliance on intra-regional trade for staples among certain small remote island countries, including Tokelau, Tuvalu, Wallis and Futuna Islands and Tonga. This is likely to be influenced by trade routes, including having limited access to extra-regional trade, directly. However, we observed the opposite for other countries: for example, there were limited intra-regional rice and wheat imports into the Federated States of Micronesia.

Overall, intra-regional trade increased after 2001, the year PICTA was signed (but not implemented). Although PICTA wasn’t implemented until 2007, it may have raised regional attention both to potential benefits of reducing trade barriers and the region as a potential market (vis a vis markets outside the region), despite our study showing limited direct impact. During this time, there have also been significant investments in trade facilitation especially for improving export standards of food crops in certain countries [42]. Overall, however, intra-regional trade in food remains a fraction of total trade. In part, this likely reflects the focus of the Pacific Island region-building exercise on extra-regional trade – similar to many other (non-EU) regional groupings [3]. It also reflects the role of long standing preferential trade agreements with extra-regional actors, particularly in relation to sugar [43].

Interest in promoting greater intra-regional trade among PICTs remained subdued throughout the 1990s, and even following PICTA there has been much greater emphasis given to promoting stronger trade links with major industrial markets where the potential for trade expansion is much greater. For example, the value of trade between the participating states of the Asia-Pacific Trade Agreement (APTA) and six PICTA countries (Fiji, Papua New Guinea, Samoa, Solomon Islands, Tonga and Vanuatu) increased steadily during the 1980s and 1990s and then grew from $121 million in 2000 to $1636 million in 2012 [44]. The majority of trade flows are exports from APTA to PICTA, but in 2012 20% was contributed by exports from PICTA to APTA members, which is notable given the size differential of member countries [44]. Many of the products exported from the region face intensive international competition and face continued pressure from substitutes, increased production from low-cost Asian producers and the inelastic demand for traditional exports [43].

The findings of this study have highlighted the growth in trade of staple foods intra-regionally, indicating an important role for Fiji (in particular) in regional food security. The increasing intra-regional contribution to imports of cereals, grains and flours over the past 20 years likely reflects a combination of re-export and local processing of staple foods, including as a result of the growing presence and scale of flour mills, particularly in Fiji. In addition, Fiji has continued to produce rice, with ongoing efforts to revitalise the rice industry [45]. This finding points to the potential for intra-regional trade to contribute to food security through increased availability and affordability of staple foods and suggests an opportunity for other commodities. For example, addressing the high cost of protein (and low consumption) in some settings, while also generating economic opportunities for development through supporting local food industries within the region [46]. This would have an additional benefit of increasing food access in times of disaster, due to overall increased regional availability (particularly given the countries in the region are mainly net-food-importers). Current high and volatile prices of staples on the international market, driven by COVID-19 and the war in Ukraine could make domestic rice production more economically viable in some PICTs. However, operationalising this potential for increased intra-regional has faced significant challenges. Several studies of intra-regional trade possibilities in the past have had no breakthrough pointing to the fact that the economic structure of PICTs and the kind of export activities they can sustain are broadly similar [26, 41].

Notable in our findings is the very limited trade in traditional staple foods, namely root crops. This likely reflects domestic production capacities and the fact that within the region there is little differential or comparative advantage in root crop production. Most PICTs still engage in root crop production and household production of traditional staple foods and vegetables is significant. For example, a 2015 agricultural survey in Tonga found that the majority (86%) of the surveyed households were active in agricultural production (cropping, livestock, fisheries, forestry or handicraft), with 37% producing for subsistence, 62% for semi-subsistence, and only 1% for commercial [47]. This indicates that majority of the households grow their own traditional food crops. Nearly all households surveyed were agricultural households, growing their own food. A similar trend of limited percentage share of households focussing on commercial (for sale) of agriculture crops is found for Samoa [48]. In Fiji, 99% of total household interviewed in rural and peri-urban areas were agricultural households, with the majority (59.4%) unpaid subsistence farmers [49].

Traditional foods are preferred in PICTs, especially in rural communities where imported foods are limited and of higher cost and there is capacity for own-production of food crops [50]. However, local consumption of traditional staple crops has been declining and across the region, food cultures have changed as people become more affluent [51]. Many of the traditional labour-intensive and time-consuming recipes are not used anymore, and younger generations have different diets from those of their parents and grandparents [52]. While the region has a very comprehensive set of food and nutrition policies, imported foods tend to be easier (cheaper and widely available) choices, and domestic staple food production is declining [53]. The limited trade in root crops also reflects the limited agricultural technologies related to storage and transport of root crops, compared to wheat and rice, which result in relatively high post-harvest losses and create disincentives for trade [54]. Trade in root crops is also challenging because sanitary and phytosanitary and technical requirements in most PICTS deters export opportunities between PICTs.

This study also identified intra-regional trade in unhealthy foods, building on previous research finding overall increases in processed food imports in Pacific Island Countries [21, 22]. This is also reflected by concerns among Pacific Island governments about unhealthy imports. For example, Tonga has implemented substantial tariff intervention to reduce unhealthy imports in recent years [55]. Overall, both intra- and extra-regional trade in unhealthy foods has been growing, and the dynamic of trade has been changing. There has been a notable shift to imports of unhealthy foods and beverages from Asia, including sugar sweetened beverages (SSBs) [22]. The Asian region has undergone major changes in agriculture and food systems, with rising food processing and export capacity [56]. Rising imports of snack foods and SSBs from Asia has also been seen in other developing regions, including Southern Africa [12].

A limitation of the study was that we were unable to ascertain the scale of retrade as a component of intra-regional trade – this is important as retrade is likely to be less beneficial to domestic economies. We were also unable to assess causation in our analysis of potential impacts of PICTA. In addition, we could not differentiate tourism as a ‘destination’ for food imports, due to a lack of information on the magnitude of consumption. In subsequent analyses that span the disruptions to trade and tourism due to COVID-19 and related measures, this may be a significant factor needed to help interpret trends in trade.

### Policy implications

The COVID-19 pandemic and associated policy measures has disrupted the production, availability and international trade of food [15, 57]. The pandemic has highlighted the long-term lack of investment by Pacific Island Governments in local food production [58] – which is also reflected in the very limited trade in local traditional foods seen in this study. For the foreseeable future, many PICs will continue to rely on own domestic agricultural production. With renewed interest in domestic agriculture following COVID-19 this study points to an opportunity for increased investments in domestic agriculture (and storage, transport and processing) to support production and trade in traditional staple foods – which are preferred and under-supplied. Further, if PICs (and donors) may consider investing on the capacity and skills of the local agricultural sectors to not only export primary produce (cassava, fresh) but also to turn it into value added products (such as cassava flour), that could address the SPS issues currently hindering the inter-PIC trade in fresh produce. At the same time, it will also diversify exports to the point that PICs may be exporting different goods to each other.

In line with the challenges outlined above, specific domestic policy opportunities relevant to enhancing healthy food availability – including through intra-regional trade – relate to investment in supply chains, as well as in resilient and affordable access to transport and internet connectivity [58]. Such policy initiatives would enhance knowledge on upcoming market opportunities and risks, while enabling affordable inter-country transportation of healthy imported foods. In addition to investment in local food production, Ministries of Agriculture across the region are highlighting the importance of increased policy focus on encouraging youth participation and entrepreneurship in agriculture [46]. This strategy would not only to help increase agricultural productivity and also reduce dependence on imported foods and thus increase food security, but also to deal with high rates of youth unemployment.

This study also raises a broader question about the potential for regional approaches to foster ‘healthier trade’. The emergence of regional trade hubs in other regions has created potential for a regional approach to improving diets and health. However, this has often occurred via health policy initiatives to improve the healthfulness of the food supply in parallel to ongoing efforts towards regional economic cooperation and liberalization, rather than via trade policy measures. In South Africa, for example, efforts to reduce salt and sugar in processed foods and to influence the nutrient composition of foods has been pursued through the Southern African Development Community (SADC) [12]. In the Pacific region, Fiji’s role as hub for intra-regional food trade means that fortified flour – an effective intervention domestically [59] – is also benefiting other countries in the region. In relation to trade in healthy food, there is potential for more food preserving and manufacturing to foster intra-regional trade in ‘Pacific’ foods that are minimally processed, for instance canned fish products, making them easier to trade and contributing to policy objectives for increased value-adding. Lifting production of locally manufactured food products for export trade will likely increase scale and affordability needed to appeal to local markets [55]. Lifting production of niche (often healthy) food products like dried fruit and juices and staple crop flours (ie cassava) is already an aim for some PICTs, such as Vanuatu [46]. However, enhancing intra-regional trade in local foods will require strengthening quality control of exports between PICTs, increasing capacity for adherence to technical and phytosanitary measures imposed by each PICTS, and investment in facilities and harmonization of requirements [60, 61].

## Conclusion

This study has documented the small but significant role of intra-regional food trade for food and nutrition security in the Pacific Island region. Fiji acts as a regional hub, and we found a heavy reliance on intra-regional trade for staples among small remote island countries. Notable in our findings is the very limited trade in root crops. Although there is a regional trade agreement, and efforts to enhance intra-regional trade have likely contributed to its growth in the region, we were unable to identify a clear impact of the main regional trade agreement on intra-regional trade. In the current context of significant food system disruption due to the COVID-19 pandemic and rising commodity prices, greater investment in traditional food export could enhance food security and nutrition in the Pacific region. More broadly, this study also echoes previous research that suggests that regional approaches offer an opportunity to foster trade in healthy foods.

## Supplementary Information

**Additional file 1:** **Supplementary 1.** Attribution of HS92 commodities for analysis of Intra-regional trade and trade agreements.

## Data Availability

All data generated or analysed during this study are included in this published article [and its supplementary information files].

## Abbreviations

APTA: Asia-Pacific Trade Agreement
MSG: Melanesian Spearhead Group
MTEC: Micronesian Trade and Economic Community
PFTD: Pacific Food Trade Database
PICTs: Pacific Island Countries and Territories
PICTA: Pacific Island Countries Trade Agreement
SADC: Southern African Development Community
SSB: Sugar Sweetened Beverage

## Footnotes

1 Pacific Island Countries and Territories include the geographic members of the Pacific Community (SPC). Detailed list here: https://www.spc.int/our-members/
