# Supplementary material for: Food trade among Pacific Island countries and territories: implications for food security and nutrition
Source: Global Health. 2022 Dec 14;18:104. doi: 10.1186/s12992-022-00891-9 (PMC9750728; doi:10.1186/s12992-022-00891-9)
Supplement: Supplementary file 1 — Additional file 1: Supplementary 1. Attribution of HS92 commodities for analysis of Intra-regional trade and trade agreements. [file 12992_2022_891_MOESM1_ESM.docx]

**Supplementary 1.** Attribution of HS92 commodities for analysis of Intra-regional trade and trade agreements.

| **HS92 Chapter definition** | **HS92 Heading definition** | **HS92 Sub-heading code** | **Extra-regional commodity** | **Intra-regional commodity** |
| --- | --- | --- | --- | --- |
| Meat and edible meat offal | Meat of sheep or goats, fresh, chilled or frozen | 020410 | 1 |  |
| Meat and edible meat offal | Meat of sheep or goats, fresh, chilled or frozen | 020421 | 1 |  |
| Meat and edible meat offal | Meat of sheep or goats, fresh, chilled or frozen | 020422 | 1 |  |
| Meat and edible meat offal | Meat of sheep or goats, fresh, chilled or frozen | 020423 | 1 |  |
| Meat and edible meat offal | Meat of sheep or goats, fresh, chilled or frozen | 020430 | 1 |  |
| Meat and edible meat offal | Meat of sheep or goats, fresh, chilled or frozen | 020441 | 1 |  |
| Meat and edible meat offal | Meat of sheep or goats, fresh, chilled or frozen | 020442 | 1 |  |
| Meat and edible meat offal | Meat of sheep or goats, fresh, chilled or frozen | 020443 | 1 |  |
| Meat and edible meat offal | Meat of sheep or goats, fresh, chilled or frozen | 020450 | 1 |  |
| Dairy products, eggs, honey, edible animal product nes | Milk and cream, concentrated or sweetened | 040210 | 1 |  |
| Dairy products, eggs, honey, edible animal product nes | Milk and cream, concentrated or sweetened | 040221 | 1 |  |
| Dairy products, eggs, honey, edible animal product nes | Milk and cream, concentrated or sweetened | 040229 | 1 |  |
| Dairy products, eggs, honey, edible animal product nes | Milk and cream, concentrated or sweetened | 040291 | 1 |  |
| Dairy products, eggs, honey, edible animal product nes | Milk and cream, concentrated or sweetened | 040299 | 1 |  |
| Dairy products, eggs, honey, edible animal product nes | Buttermilk, cream, yogurt etc | 040310 | 1 |  |
| Dairy products, eggs, honey, edible animal product nes | Buttermilk, cream, yogurt etc | 040390 | 1 |  |
| Dairy products, eggs, honey, edible animal product nes | Whey, natural milk products nes | 040410 | 1 |  |
| Dairy products, eggs, honey, edible animal product nes | Whey, natural milk products nes | 040490 | 1 |  |
| Dairy products, eggs, honey, edible animal product nes | Butter and other fats and oils derived from milk | 040500 | 1 |  |
| Dairy products, eggs, honey, edible animal product nes | Cheese and curd | 040610 | 1 |  |
| Dairy products, eggs, honey, edible animal product nes | Cheese and curd | 040620 | 1 |  |
| Dairy products, eggs, honey, edible animal product nes | Cheese and curd | 040630 | 1 |  |
| Dairy products, eggs, honey, edible animal product nes | Cheese and curd | 040640 | 1 |  |
| Dairy products, eggs, honey, edible animal product nes | Cheese and curd | 040690 | 1 |  |
| Edible vegetables and certain roots and tubers | Potatoes, fresh or chilled | 070190 | 1 |  |
| Edible vegetables and certain roots and tubers | Onions, shallots, garlic, leeks, etc. fresh or chille | 070310 | 1 |  |
| Edible vegetables and certain roots and tubers | Onions, shallots, garlic, leeks, etc. fresh or chille | 070320 | 1 |  |
| Edible vegetables and certain roots and tubers | Onions, shallots, garlic, leeks, etc. fresh or chille | 070390 | 1 |  |
| Edible vegetables and certain roots and tubers | Cabbage, cauliflower, kohlrabi & kale, fresh, chilled | 070410 | 1 |  |
| Edible vegetables and certain roots and tubers | Cabbage, cauliflower, kohlrabi & kale, fresh, chilled | 070420 | 1 |  |
| Edible vegetables and certain roots and tubers | Manioc, rrowroot, salep etc, fresh, dried, sago pith | 071410 |  | 1 |
| Edible vegetables and certain roots and tubers | Manioc, rrowroot, salep etc, fresh, dried, sago pith | 071420 |  | 1 |
| Edible vegetables and certain roots and tubers | Manioc, rrowroot, salep etc, fresh, dried, sago pith | 071490 |  | 1 |
| Edible fruit, nuts, peel of citrus fruit, melons | Coconuts, Brazil nuts and cashew nuts, fresh or dried | 080110 |  | 1 |
| Edible fruit, nuts, peel of citrus fruit, melons | Coconuts, Brazil nuts and cashew nuts, fresh or dried | 080120 | 1 |  |
| Edible fruit, nuts, peel of citrus fruit, melons | Coconuts, Brazil nuts and cashew nuts, fresh or dried | 080130 | 1 |  |
| Edible fruit, nuts, peel of citrus fruit, melons | Nuts except coconut, brazil & cashew, fresh or dried | 080211 | 1 |  |
| Edible fruit, nuts, peel of citrus fruit, melons | Nuts except coconut, brazil & cashew, fresh or dried | 080212 | 1 |  |
| Edible fruit, nuts, peel of citrus fruit, melons | Nuts except coconut, brazil & cashew, fresh or dried | 080221 | 1 |  |
| Edible fruit, nuts, peel of citrus fruit, melons | Nuts except coconut, brazil & cashew, fresh or dried | 080222 | 1 |  |
| Edible fruit, nuts, peel of citrus fruit, melons | Nuts except coconut, brazil & cashew, fresh or dried | 080231 | 1 |  |
| Edible fruit, nuts, peel of citrus fruit, melons | Nuts except coconut, brazil & cashew, fresh or dried | 080232 | 1 |  |
| Edible fruit, nuts, peel of citrus fruit, melons | Nuts except coconut, brazil & cashew, fresh or dried | 080240 | 1 |  |
| Edible fruit, nuts, peel of citrus fruit, melons | Nuts except coconut, brazil & cashew, fresh or dried | 080250 | 1 |  |
| Edible fruit, nuts, peel of citrus fruit, melons | Bananas, including plantains, fresh or dried | 080300 |  | 1 |
| Edible fruit, nuts, peel of citrus fruit, melons | Grapes, fresh or dried | 080610 | 1 |  |
| Edible fruit, nuts, peel of citrus fruit, melons | Grapes, fresh or dried | 080620 | 1 |  |
| Edible fruit, nuts, peel of citrus fruit, melons | Apples, pears and quinces, fresh | 080810 | 1 |  |
| Edible fruit, nuts, peel of citrus fruit, melons | Apples, pears and quinces, fresh | 080820 | 1 |  |
| Edible fruit, nuts, peel of citrus fruit, melons | Stone fruit, fresh (apricot, cherry, plum, peach, etc | 080910 | 1 |  |
| Edible fruit, nuts, peel of citrus fruit, melons | Stone fruit, fresh (apricot, cherry, plum, peach, etc | 080920 | 1 |  |
| Edible fruit, nuts, peel of citrus fruit, melons | Stone fruit, fresh (apricot, cherry, plum, peach, etc | 080930 | 1 |  |
| Edible fruit, nuts, peel of citrus fruit, melons | Stone fruit, fresh (apricot, cherry, plum, peach, etc | 080940 | 1 |  |
| Edible fruit, nuts, peel of citrus fruit, melons | Fruits nes, fresh | 081010 | 1 |  |
| Edible fruit, nuts, peel of citrus fruit, melons | Fruits nes, fresh | 081020 | 1 |  |
| Edible fruit, nuts, peel of citrus fruit, melons | Fruits nes, fresh | 081030 | 1 |  |
| Edible fruit, nuts, peel of citrus fruit, melons | Fruits nes, fresh | 081040 | 1 |  |
| Edible fruit, nuts, peel of citrus fruit, melons | Fruits nes, fresh | 081090 | 1 |  |
| Edible fruit, nuts, peel of citrus fruit, melons | Fruits and nuts, uncooked boiled or steamed, frozen | 081110 | 1 |  |
| Edible fruit, nuts, peel of citrus fruit, melons | Fruits and nuts, uncooked boiled or steamed, frozen | 081120 | 1 |  |
| Edible fruit, nuts, peel of citrus fruit, melons | Fruits and nuts, uncooked boiled or steamed, frozen | 081190 | 1 |  |
| Edible fruit, nuts, peel of citrus fruit, melons | Fruits, nuts provisionally preserved, not ready to ea | 081210 | 1 |  |
| Edible fruit, nuts, peel of citrus fruit, melons | Fruits, nuts provisionally preserved, not ready to ea | 081220 | 1 |  |
| Edible fruit, nuts, peel of citrus fruit, melons | Fruit, dried, nes, dried fruit and nut mixtures | 081310 | 1 |  |
| Edible fruit, nuts, peel of citrus fruit, melons | Fruit, dried, nes, dried fruit and nut mixtures | 081320 | 1 |  |
| Edible fruit, nuts, peel of citrus fruit, melons | Fruit, dried, nes, dried fruit and nut mixtures | 081330 | 1 |  |
| Cereals | Wheat and meslin | 100110 | 1 |  |
| Cereals | Wheat and meslin | 100190 | 1 |  |
| Cereals | Rye | 100200 | 1 |  |
| Cereals | Barley | 100300 | 1 |  |
| Cereals | Oats | 100400 | 1 |  |
| Cereals | Maize (corn) | 100590 | 1 |  |
| Cereals | Maize (corn) | 100590 | 1 |  |
| Cereals | Rice | 100610 | 1 |  |
| Cereals | Rice | 100620 | 1 |  |
| Cereals | Rice | 100630 | 1 |  |
| Cereals | Rice | 100640 | 1 |  |
| Cereals | Buckwheat, millet and canary seed, other cereals | 100820 | 1 |  |
| Cereals | Buckwheat, millet and canary seed, other cereals | 100890 | 1 |  |
| Milling products, malt, starches, inulin, wheat glute | Wheat or meslin flour | 110100 | 1 |  |
| Milling products, malt, starches, inulin, wheat glute | Cereal flours other than of wheat or meslin | 110210 | 1 |  |
| Milling products, malt, starches, inulin, wheat glute | Cereal flours other than of wheat or meslin | 110220 | 1 |  |
| Milling products, malt, starches, inulin, wheat glute | Cereal flours other than of wheat or meslin | 110230 | 1 |  |
| Milling products, malt, starches, inulin, wheat glute | Cereal flours other than of wheat or meslin | 110290 | 1 |  |
| Milling products, malt, starches, inulin, wheat glute | Cereal grouts, meal and pellets | 110311 | 1 |  |
| Milling products, malt, starches, inulin, wheat glute | Cereal grouts, meal and pellets | 110312 | 1 |  |
| Milling products, malt, starches, inulin, wheat glute | Cereal grouts, meal and pellets | 110313 | 1 |  |
| Milling products, malt, starches, inulin, wheat glute | Cereal grouts, meal and pellets | 110314 | 1 |  |
| Milling products, malt, starches, inulin, wheat glute | Cereal grouts, meal and pellets | 110319 | 1 |  |
| Milling products, malt, starches, inulin, wheat glute | Cereal grouts, meal and pellets | 110321 | 1 |  |
| Milling products, malt, starches, inulin, wheat glute | Cereal grouts, meal and pellets | 110329 | 1 |  |
| Milling products, malt, starches, inulin, wheat glute | Worked cereal grains except flour, groat, meal, pelle | 110411 | 1 |  |
| Milling products, malt, starches, inulin, wheat glute | Worked cereal grains except flour, groat, meal, pelle | 110412 | 1 |  |
| Milling products, malt, starches, inulin, wheat glute | Worked cereal grains except flour, groat, meal, pelle | 110419 | 1 |  |
| Milling products, malt, starches, inulin, wheat glute | Worked cereal grains except flour, groat, meal, pelle | 110421 | 1 |  |
| Milling products, malt, starches, inulin, wheat glute | Worked cereal grains except flour, groat, meal, pelle | 110422 | 1 |  |
| Milling products, malt, starches, inulin, wheat glute | Worked cereal grains except flour, groat, meal, pelle | 110423 | 1 |  |
| Milling products, malt, starches, inulin, wheat glute | Worked cereal grains except flour, groat, meal, pelle | 110429 | 1 |  |
| Milling products, malt, starches, inulin, wheat glute | Worked cereal grains except flour, groat, meal, pelle | 110430 | 1 |  |
| Milling products, malt, starches, inulin, wheat glute | Potato flour, meal, flakes, etc | 110510 | 1 |  |
| Milling products, malt, starches, inulin, wheat glute | Potato flour, meal, flakes, etc | 110520 | 1 |  |
| Milling products, malt, starches, inulin, wheat glute | Flour and meal of legumes, roots, tubers, nuts, citru | 110610 |  | 1 |
| Milling products, malt, starches, inulin, wheat glute | Flour and meal of legumes, roots, tubers, nuts, citru | 110620 |  | 1 |
| Milling products, malt, starches, inulin, wheat glute | Flour and meal of legumes, roots, tubers, nuts, citru | 110630 |  | 1 |
| Milling products, malt, starches, inulin, wheat glute | Malt | 110710 | 1 |  |
| Milling products, malt, starches, inulin, wheat glute | Malt | 110720 | 1 |  |
| Milling products, malt, starches, inulin, wheat glute | Starches, inulin | 110811 | 1 |  |
| Milling products, malt, starches, inulin, wheat glute | Starches, inulin | 110812 | 1 |  |
| Milling products, malt, starches, inulin, wheat glute | Starches, inulin | 110813 | 1 |  |
| Milling products, malt, starches, inulin, wheat glute | Wheat gluten | 110900 | 1 |  |
| Oil seed, oleagic fruits, grain, seed, fruit, etc, ne | Soya beans | 120100 | 1 |  |
| Oil seed, oleagic fruits, grain, seed, fruit, etc, ne | Copra | 120300 |  | 1 |
| Oil seed, oleagic fruits, grain, seed, fruit, etc, ne | Linseed | 120400 | 1 |  |
| Oil seed, oleagic fruits, grain, seed, fruit, etc, ne | Rape or colza seeds | 120500 | 1 |  |
| Oil seed, oleagic fruits, grain, seed, fruit, etc, ne | Sunflower seeds | 120600 | 1 |  |
| Oil seed, oleagic fruits, grain, seed, fruit, etc, ne | Oil seeds and oleaginous fruits nes | 120740 | 1 |  |
| Oil seed, oleagic fruits, grain, seed, fruit, etc, ne | Oil seeds and oleaginous fruits nes | 120750 | 1 |  |
| Oil seed, oleagic fruits, grain, seed, fruit, etc, ne | Oil seeds and oleaginous fruits nes | 120760 | 1 |  |
| Oil seed, oleagic fruits, grain, seed, fruit, etc, ne | Oil seeds and oleaginous fruits nes | 120791 | 1 |  |
| Oil seed, oleagic fruits, grain, seed, fruit, etc, ne | Hop cones, fresh or dried, whether or not ground…. | 121010 | 1 |  |
| Oil seed, oleagic fruits, grain, seed, fruit, etc, ne | Hop cones, fresh or dried, whether or not ground…. | 121020 | 1 |  |
| Animal,vegetable fats and oils, cleavage products, et | Soya-bean oil, fractions, not chemically modified | 150710 | 1 |  |
| Animal,vegetable fats and oils, cleavage products, et | Soya-bean oil, fractions, not chemically modified | 150790 | 1 |  |
| Animal,vegetable fats and oils, cleavage products, et | Ground-nut oil, fractions, not chemically modified | 150810 | 1 |  |
| Animal,vegetable fats and oils, cleavage products, et | Ground-nut oil, fractions, not chemically modified | 150890 | 1 |  |
| Animal,vegetable fats and oils, cleavage products, et | Olive oil and its fractions, not chemically modified | 150910 | 1 |  |
| Animal,vegetable fats and oils, cleavage products, et | Olive oil and its fractions, not chemically modified | 150990 | 1 |  |
| Animal,vegetable fats and oils, cleavage products, et | Olive oil, fractions, blends, not chemically modified | 151000 | 1 |  |
| Animal,vegetable fats and oils, cleavage products, et | Safflower, sunflower and cotton-seed oil, fractions | 151211 | 1 |  |
| Animal,vegetable fats and oils, cleavage products, et | Safflower, sunflower and cotton-seed oil, fractions | 151219 | 1 |  |
| Animal,vegetable fats and oils, cleavage products, et | Safflower, sunflower and cotton-seed oil, fractions | 151221 | 1 |  |
| Animal,vegetable fats and oils, cleavage products, et | Safflower, sunflower and cotton-seed oil, fractions | 151229 | 1 |  |
| Animal,vegetable fats and oils, cleavage products, et | Rape, colza, mustard oil, fractions, simply refined | 151410 | 1 |  |
| Animal,vegetable fats and oils, cleavage products, et | Margarine, edible animal or veg oil preparations nes | 151710 | 1 |  |
| Sugars and sugar confectionery | Solid cane or beet sugar and chemically pure sucrose | 170111 | 1 |  |
| Sugars and sugar confectionery | Solid cane or beet sugar and chemically pure sucrose | 170112 | 1 |  |
| Sugars and sugar confectionery | Solid cane or beet sugar and chemically pure sucrose | 170191 | 1 |  |
| Sugars and sugar confectionery | Solid cane or beet sugar and chemically pure sucrose | 170199 | 1 |  |
| Sugars and sugar confectionery | Sugars nes, lactose, fructose, glucose, maple syrup | 170210 | 1 |  |
| Sugars and sugar confectionery | Sugars nes, lactose, fructose, glucose, maple syrup | 170220 | 1 |  |
| Sugars and sugar confectionery | Sugars nes, lactose, fructose, glucose, maple syrup | 170230 | 1 |  |
| Sugars and sugar confectionery | Sugars nes, lactose, fructose, glucose, maple syrup | 170240 | 1 |  |
| Sugars and sugar confectionery | Sugars nes, lactose, fructose, glucose, maple syrup | 170250 | 1 |  |
| Sugars and sugar confectionery | Sugars nes, lactose, fructose, glucose, maple syrup | 170260 | 1 |  |
| Sugars and sugar confectionery | Sugars nes, lactose, fructose, glucose, maple syrup | 170290 | 1 |  |
| Sugars and sugar confectionery | Molasses from the extraction or refining of sugar | 170310 | 1 |  |
| Sugars and sugar confectionery | Molasses from the extraction or refining of sugar | 170390 | 1 |  |
| Sugars and sugar confectionery | Sugar confectionery, non-cocoa, white chocolate | 170410 | 1 |  |
| Sugars and sugar confectionery | Sugar confectionery, non-cocoa, white chocolate | 170490 | 1 |  |
| Cereal, flour, starch, milk preparations and products | Pasta, couscous, etc. | 190211 | 1 |  |
| Cereal, flour, starch, milk preparations and products | Pasta, couscous, etc. | 190219 | 1 |  |
| Cereal, flour, starch, milk preparations and products | Pasta, couscous, etc. | 190220 | 1 |  |
| Cereal, flour, starch, milk preparations and products | Pasta, couscous, etc. | 190230 | 1 |  |
| Cereal, flour, starch, milk preparations and products | Pasta, couscous, etc. | 190240 | 1 |  |
